# WARMTH Is “Warming Up”

**DOI:** 10.1055/s-0044-1795167

**Published:** 2024-12-12

**Authors:** Kalevi Kairemo

**Affiliations:** 1 Docrates Cancer Center, Helsinki, Finland

The year 2024 has been a real success story for the World Association of Radiopharmaceutical and Molecular Therapy (WARMTH). The first highlight was the publication of the third WARMTH trial describing targeted alpha therapy using Ac-225-PSMA-617 in *The Lancet Oncology* in February. 1 In this retrospective study with 488 patients from four continents, Sathekge et al report 1 an excellent outcome. More than half of the advanced stage patients responded and limited adverse effects were observed, and multiple factors affecting response could be identified. Additionally, I was requested to provide a commentary for *The Lancet Oncology* . 2 The WARMTH has conducted altogether three major global trials, which were published in high-ranked journals. The first two were retrospective trials using Lu-177-PSMA-617 (the same compound as Pluvicto), both published in the *European Journal of Nuclear Medicine and Molecular Imaging* . In these studies, more than 400 patients were treated and factors predicting outcome and excellent results could be confirmed before official registration trials, using real-world evidence data.

The main congress of the WARMTH was held this year in Muscat, Oman, from February 8 to 12. This 19th International Conference of Radiopharmaceutical Therapy (ICRT) was a well-attended congress with participant representation from all continents and a wide range of multidisciplinary fields. This was a successful meeting and the abstracts of the congress were published in the *World Journal of Nuclear Medicine (WJNM)* . The World Theranostics Day (March 31) was celebrated on April 3 as an online meeting with two well-known speakers, Drs. Hossein Jadvar from Los Angeles, United States, and Andrew M. Scott from Melbourne, Australia. This live online event was moderated from Helsinki, Finland, with technical support from California and it gathered almost 300 participants worldwide.

The WARMTH was present at the Society of Nuclear Medicine and Molecular Imaging (SNMMI) meeting in Toronto, Canada, in June by arranging a joint session for radionuclide therapy. In the Asia and Oceania Congress of Nuclear Medicine and Biology (AOCNMB) in Bali, Indonesia, the WARMTH arranged an own International Symposium of Radiopharmaceutical Therapy (ISRT) with two sessions. The AOCNMB takes place every 3 years. Thus, the Indonesian congress in September was the first fully live event in 6 years. The WARMTH had a theragnostic session in iPET conference in Vienna, Austria, in October. In this iPET meeting arranged by the International Atomic Energy Agency (IAEA), the WARMTH had strong participation with an own session and panel. This event was fully live for the first time in 7 years and there were participants from 103 countries. The meeting culminated in the WARMTH and the IAEA “Practical Arrangements” signing ceremony. The WARMTH has had a very strong connection with IAEA and its member states in promoting the medical use of radionuclides globally, because the founder of the WARMTH, Dr. Ajit Padhy, was earlier an IAEA employee. The WARMTH will continue with this important task and therefore strengthened cooperation with the IAEA. More importantly, we in the WARMTH used the relatively novel paradigm of “Thera(g)nostics” from the very beginning. During 2024, there were some smaller WARMTH-associated events. The most important ISRT was held in Lagos, Nigeria, in July, which included a practical workshop.

The WARMTH initiatives are based on the premise of global growth of nuclear medicine therapies in all countries. This year, we have signed memorandums of understanding with various international communities including the SNMMI and The International Centers for Precision Oncology Foundation (ICPO). These are meant to extend the WARMTH involvement and influence and continue to provide expert networking on the global platform.

The year 2025 is already being planned with many collaborative meetings from January. The main congress, the 20th ICRT will be held from November 6 to 9, 2025 in Cyprus, which will be arranged in collaboration with the Cyprus Society of Nuclear Medicine. An ISRT will be held from April 10 to 12, 2025 in Baku, Azerbaijan, together with the Azerbaijan Society of Nuclear Medicine. The WARMTH will be present in the ALASBIMN (Latin American Societies) meeting in March in Quintana Roo, Mexico, SNMMI meeting in June in New Orleans, United States, and in the European (EANM) meeting in October in Barcelona, Spain. The 13th Conference for Targeted Alpha Therapy will be held in May 2025 in Oslo. The WARMTH is planning to have an own session in this event, which takes place every 2 years.

Additionally, one of the WARMTH governing body members has been elected as President of the World Federation of Nuclear Medicine and Biology (WFNMB). We, in the WARMTH, look forward to a great collaboration between the WARMTH and the WFNMB.

The WARMTH, as a voluntary nonprofit organization of enthusiastic individuals, is willing to advance science and education of radiopharmaceutical therapies for the benefit of public health and humanity and is willing to work toward worldwide access to these theragnostic procedures.

Helsinki, November 4, 2024

Kalevi Kairemo

President, WARMTH
